# Ingestion of a Pre-bedtime Protein Containing Beverage Prevents Overnight Induced Negative Whole Body Protein Balance in Healthy Middle-Aged Men: A Randomized Trial

**DOI:** 10.3389/fnut.2019.00181

**Published:** 2019-11-29

**Authors:** Leonidas G. Karagounis, Maurice Beaumont, Laurence Donato-Capel, Jean-Philippe Godin, Anne-France Kapp, Dimitrios Draganidis, Stéphane Pinaud, Jacques Vuichoud, Maya Shevlyakova, Koraljka Rade-Kukic, Denis Breuillé

**Affiliations:** ^1^Nestlé Research, Lausanne, Switzerland; ^2^Institute of Social and Preventive Medicine, University of Bern, Bern, Switzerland; ^3^Translation Research, Nestlé Health Science, Lausanne, Switzerland

**Keywords:** milk protein, aging, sleep, muscle, protein balance

## Abstract

Age related muscle wasting leads to overall reductions of lean body mass, reduced muscle strength, and muscle function resulting in compromised quality of life. Utilizing novel nutritional strategies to attenuate such losses is of great importance in elderly individuals. We aimed to test if a complete dietary supplement containing 25 g of milk proteins and ingested in the evening before bed would improve protein metabolism in terms of whole body protein balance over a 10 h overnight period following ingestion of the test drink in healthy middle-aged male subjects. In addition we also assessed the rates of muscle protein synthesis during the second half of the night in order to see if previously reported extended amino acidemia during sleep results in increased rates of muscle protein synthesis. Seventeen healthy middle-aged male subjects (59.4 ± 3.2 year) consumed a dietary supplement drink at 21:00 containing either 25 g milk protein concentrate, 25 g maltodextrin, 7.75 g canola oil (treatment group), or an isocaloric protein void drink (placebo group). Muscle protein synthesis was assessed from a muscle biopsy following the continuous intravenous infusion of ^13^C-phenylalanine for 5 h (from 03:00 to 08:00). Whole body protein balance was greater in the treatment group (−0.13 ± 11.30 g prot/10 h) compared to placebo (−12.22 ± 6.91 g prot/10 h) (*P* ≤ 0.01). In contrast, no changes were observed on rates of muscle protein synthesis during the second half of the night. Ingestion of a dietary supplement containing 25 g of milk proteins significantly reduced the negative protein balance observed during the night. Therefore, pre-bedtime protein ingestion may attenuate overnight losses of lean tissue in healthy elderly men. Despite increases in aminoacidemia during the second part of the night, no changes were observed in the rates of muscle protein synthesis during this time.

**Clinical Trial Registration:** www.ClinicalTrials.gov, identifier: NCT02041143.

## Introduction

Skeletal muscle is the most abundant tissue in the human body and plays a key role in both locomotion and metabolic health and it is therefore important to maintain healthy muscle mass and function as we age (1). Two triggers known to stimulate adaptations in skeletal muscle include nutrition (macro- and micro-nutrients) (2–5) and contractile activity (weight loading and unloading) (1, 6–9). Repeated exposure to such stimuli over time initiate changes at the molecular level allowing skeletal muscle to adapt and alter its profile to meet the demands of its new environment (10). This regulation of skeletal muscle protein turnover is primarily coordinated by changes in the rates of both muscle protein synthesis (MPS) and muscle protein breakdown (MPB). However, with the progression of age, the rate of muscle protein turnover and especially the lower postprandial MPS response may contribute to a reduction in muscle mass and function (11). Specifically, at a population level it has been demonstrated that after the fifth decade of life muscle mass is reduced by ~1% per year (12). This accumulated loss of muscle mass over time may lead to mobility limitations in older individuals (13).

Fasting, such as that observed during overnight sleep, results in negative muscle protein balance as a result of both, reduced rates of muscle protein synthesis and increased rates of muscle protein breakdown (9, 14–16). Nutritional strategies aimed at maintaining and improving lean tissue body composition may be beneficial for elderly populations who are at risk of muscle atrophy and accelerated muscle loss as observed under sarcopenic conditions. Therefore, attenuating the overnight losses of lean tissue mass (including muscle) would potentially be beneficial in helping maintain lean tissue/muscle mass in the elderly. Several bodies of work have addressed this proof of concept in young, healthy, physically active subjects which have been reviewed in recent work by Trommelen and Van Loon (17). Specifically, Res et al., reported that 25 g post-exercise (21:30) whey protein ingestion followed by a second 40 g bolus ingestion of casein at 23:30 (before bed time) resulted in increased muscle protein balance over an 8-h period in young healthy male subjects (18). In addition, the authors reported that plasma amino acid concentrations remained elevated throughout the sleep duration highlighting a potential extension in the nutrient driven anabolic state. More recently, Kouw et al. (19) showed that the ingestion of 40 g protein before sleep increased myofibrillar protein synthesis during overnight sleep. This effect was not observed after ingestion of 20 g protein. However, what is not clear is whether or not the increased whole body protein balance observed in the morning was due to an extension in the postprandial anabolic window beyond that normally observed (within 3–5 h postprandialy). Moreover, it is not known if such a benefit would be observed in older individuals following the ingestion of a complete oral nutritional supplement containing 25 g of dairy protein together with carbohydrate and fat.

Therefore, the aim of the present study was to investigate if whole body nitrogen balance would be improved after ingestion of a complete oral nutritional supplement (25 g milk protein, 25 g maltodextrin, and 7.5 g canola oil) in healthy elderly individuals. In addition we aimed to identify if there is an extension to the anabolic window of opportunity for muscle protein synthesis beyond the commonly reported 3–5 h postprandial window due to increased amino acid availability.

## Subjects and Methods

### Subject Characteristics

**Figure 1:**
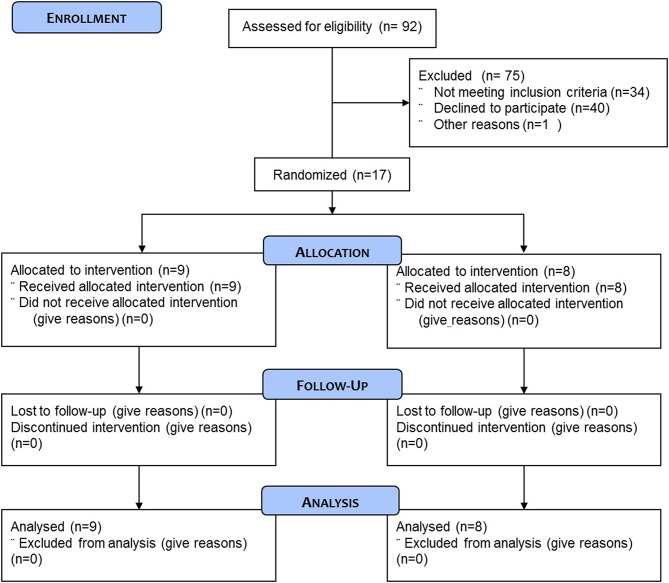
CONSORT flow diagram of the study.

A total of 17 healthy recreationally active men aged 59.4 ± 3.2 year (range: 55–66 year) were selected to participate in this study (Figure 1). Subjects were recruited locally, through a recruitment agency (TrialReach, Nyon, Switzerland). Inclusion criteria were healthy males aged 50–70, with a BMI between 19.0 and 27.0 kg/m^2^. Main exclusion criteria were any cardiovascular disease including arterial hypertension, any clotting disorders or any chronic intake of medications jeopardizing clotting, participation in any special diet (vegan, vegetarian, high protein intake) or weight loss program, food allergies, medication for chronic disease or any people following a strict exercise regime in order to either lose weight, gain muscle or reach competition standards for a chosen sport. The study was approved by the Commission Cantonale d'Ethique (VD) pour la Recherche sur l'Etre Humain, Switzerland, under the reference 423/13.

Subjects were randomly assigned to either the protein containing supplement (PRO: *n* = 9) or an isocaloric protein void placebo supplement (PLA: *n* = 8) experimental group. One individual in the PLA experiment group had to be excluded because it was not possible to obtain the muscle biopsy. Subject characteristics are presented in Table 1. All subjects were fully informed on the nature and possible risks of the experimental procedures before their written informed consent was obtained. Before taking part, all subjects underwent routine medical screening and eligible participants all gave their informed signed consent to take part in the study and were aware that they were free to withdraw from the experiment at any point.

**Table 1:** Baseline characteristics of participants.

	**Placebo (*n* = 8)**	**Treatment (*n* = 9)**	***p* (*t*-test)**
Age (years)	60.4 ± 3.02	57.9 ± 2.93	0.11
Height (cm)	181 ± 7.33	178 ± 7.38	0.55
Weight (kg)	79.6 ± 9.59	76.1 ± 9.85	0.46
BMI (kg/m^2^)	24.4 ± 1.47	23.8 ± 1.64	0.48
Diastolic blood pressure (mmHg)	82.6 ± 5.10	80 ± 8.40	0.44
Systolic blood pressure (mmHg)	135 ± 5.98	125 ± 8.82	0.03^*^

*Values are means ± SD. For comparison of numerical values, statistics were obtained with the use of Student's t-test*.

* *Different from controls (P < 0.05)*.

### Experimental Design

**Figure 2:**
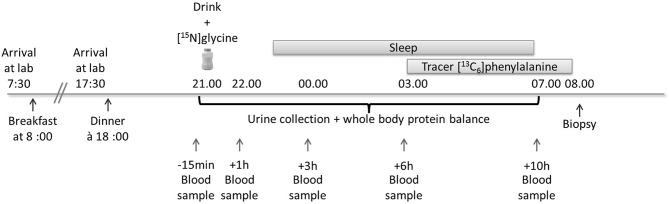
Study overview for the measurement of the effect of pre-bedtime dietary protein intake and its effect on whole body protein balance and rates of muscle protein synthesis. A drink containing 25 g of milk protein concentrate was ingested at 21:00 h. For the measurement of muscle protein synthesis a tracer ([^13^C_6_]phenylalanine) infusion was commenced at 03:00 h and a single muscle biopsy was obtained 5 h later at 08:00 h.

A detailed schema outlining the study procedure is described in Figure 2.

Eligible subjects reported to the Nestlé Metabolic Unit in the morning of the experimental day at 07:30 having fasted from 22:00 the preceding evening. A fasted blood sample was obtained in order to measure blood clotting parameters (prothrombin time, activated partial thromboplastin time, and fibrinogen). Each subject was provided with a standardized breakfast which was consumed on site. Subsequently, each subject was provided with a standardized lunch and advised to consume it at 12:00. All subjects reported back to the Nestlé Metabolic Unit at 17:30 and were provided with a standardized dinner which was consumed at 18:00. Subjects were not controlled for snacks out of the laboratory but they were asked to refrain from consuming protein supplements in the 15 days preceding the testing session. The total amount of protein in each of the standardized meals was defined as 1.2 g protein per kg of body weight. For practical reasons, 1 meal portion was designed to contain 61.15 g of protein (chicken breast) and subjects were given 1, 1.25, 1.5, or 1.75 portions as a function of their body weight. Following completion of the dinner meal, two catheters were inserted into an antecubital vein.

Prior to the ingestion of the tracer at 21:00 subjects voided their bladder. Subsequently, a blood sample was obtained and used for baseline measurements of plasma amino acids, glucose and insulin. Following this and 15 min before the commencement of the experimental trial, each subject ingested the intervention supplement (at 21:00) containing 200 mg of [^15^N]glycine stable isotope tracer (Euriso-top, St. Aubin, France). The intervention product contained either 3.1% canola oil, 10% maltodextrin DE21, and 10% milk protein concentrate (test product) or 3.1% canola oil, and 20% maltodextrin DE21 (placebo). Both products were isocaloric with a total calorie content of 270 kcal per drink (test product). Each subject in each group was required to consume 250 g of either the study test product or placebo. Each subject remained in a resting condition for 2 h before going to bed (23:00 h) for an 8-h sleep period.

At 03:00 the plasma phenylalanine pools were primed with a single intravenous dose of [^13^C_6_]phenylalanine (2.0 μmol/kg). Thereafter, a continuous stable isotope infusion of [^13^C_6_]phenylalanine (Euriso-top, St. Aubin, France) was initiated at an infusion rate of 0.050 μmol kg^−1^ min^−1^ for 300 min. At the end of the infusion (*t* = 8.00 h) a muscle biopsy was obtained from the vastus lateralis muscle under local anesthesia (see Figure 2). During the infusion of the [^13^C_6_]phenylalanine, arterialized blood samples were obtained to confirm steady state conditions.

Blood samples were obtained at specific time points throughout the night (−15 min before drink ingestion and 1, 3, 6, 7, 10, and 11 h following drink ingestion) without waking the subjects. Urine was collected once the subjects had woken or during toilet breaks during the night. Subjects woke up at 07:00. Following the muscle biopsy procedure, each subject was provided with a standard breakfast.

### Muscle Biopsy

Muscle biopsies were performed according to the Bergstrom methodology. Briefly, local anesthesia [2–3 ml of 1% xylocain (rapidocain^®^)] was administered to the skin, subcutaneous tissue and fascia of the vastus lateralis in preparation for muscle sampling. A muscle biopsy sample (100–150 mg) of the Vastus lateralis was obtained using a 5 mm Bergstrom needle with manual suction by an experienced practitioner. The muscle sample was then washed and immediately frozen in liquid nitrogen and stored at −80°C for further analysis.

### Whole Body Protein Balance

Whole body protein turnover was calculated using the end-product method as described previously (20). The ^15^N isotopic enrichments of urinary ammonia and urea samples were determined in duplicate after isolation of both components by cation exchange resin (Bio-Rad AG, Reinach, Switzerland) and measured by isotope ratio mass spectrometry (Delta V coupled to an elemental analyzer, Thermo Fischer, Bremen, Germany). Concentrations of urinary urea and creatinine, the major nitrogen containing metabolites in urine, were measured by an automated analyzer (Cobas, Hoffmann- La Roche, Switzerland). Isotopic enrichments were expressed as TTR (tracer to tracee ratio) corrected from baseline isotopic enrichment. Whole body nitrogen turnover (Q) was calculated as Q (mg N/kg/h) = *d*/Ei/BW/time; where *d* is the dose of oral [^15^N]glycine in g, Ei is the isotopic enrichment of ^15^N ammonia, *t* is the time of urine collection (i.e., 10 h) and BW is the body weight expressed in kg (21). Whole body protein synthesis (S) and whole body protein breakdown B were then calculated from the expression Q = S + E = B + I; where E is the excretion of nitrogen estimated as the sum of urea and creatinine excretion plus other losses (i.e., fecal and miscellaneous). Protein synthesis and protein breakdown were normalized by body weight (BW) and finally expressed as mg protein/kg BW/h (using 6.25 as coefficient to transform nitrogen to protein fluxes).

### Muscle Protein Synthesis

Mixed muscle fractional synthesis rate (FSR) were based on the single biopsy approach (22, 23). FSR (expressed in %/h) was calculated using the standard precursor-product equation (validated for a single muscle biopsy approach) as described by Burd et al. (23).

$$\begin{array}{l} \text{FSR}\left(\%/\text{h}\right)=\left({\text{E}}_{\text{p}2}-{\text{E}}_{\text{p}1}\right)/{\text{E}}_{\text{ic}}/\text{t}\times \;100; \end{array}$$

Where, E_p2_ and E_p1_ are the ^13^C isotopic enrichment of Phe from protein bound in muscle (from the muscle biopsy) and Phe from plasma protein, respectively. E_ic_ is the mean intracellular phenylalanine enrichment from the biopsy and t is the tracer incorporation time.

### Sample Collection, Processing, and Analysis for FSR Determination

Blood samples were collected in pre-chilled tubes containing EDTA. Plasma was separated by centrifugation and then stored at −80°C until analysis. Muscle samples were rinsed in ice-cold saline solution immediately after collection, then cleared from fat tissues and finally frozen in liquid nitrogen.

To determine plasma ^13^C Phe isotopic enrichment expressed as tracer-to-tracee ratio (TTR), plasma was precipitated with acetonitrile and free amino acids were derivatized by preparing propyl-chloroformate derivatives and analyzed using Phenomenex ZB-AAA column (15 m × 0.25 mm, 0.1 μm I.D film thickness column, from Brechbuller, Switzerland). Plasma Phe TTR were monitored by gas chromatography/mass spectrometry (GC-MS, MSD 5973 System, Hewlett Packard).

^13^C isotopic enrichment of Phe from plasma protein and muscle protein (biopsy samples) were determined using a gas chromatography-combustion-isotope ratio mass spectrometry (Delta V Hyphenated to GC system from Thermo Fischer, Bremen, Germany). Isotopic data (^13^C/^12^C ratio) were expressed in delta per mil value (δ^13^C, ‰). The pellets containing muscle and plasma proteins was washed and then hydrolyzed with 6 N HCL at 110°C for 24 h. Amino acids obtained were then purified on cation exchange chromatography (Dowex 50W-X8-200, Bio-Rad Laboratories), then derivatized to get N-acetyl-n-propyl ester derivative (NAP) amino acid and analyzed on DB5-MS column (30 m × 0.25 mm; 0.25 μm I.D film thickness column, from Agilent, Germany).

For FSR calculation, in this study, background ^13^C-Phe enrichment of mixed plasma proteins for the initial isotopic enrichment as described by Burd et al. and intracellular ^13^C-Phe (as precursor pool) were used. We found that the plasma protein measured before administration of the tracer was δ^13^C = −32.49 ± 0.52 ‰ (*n* = 17, expressed as mean ± SD) showing a good repeatability between subjects. Like in most of the studies with ^13^C-Phe as tracer, we used muscle intracellular free ^13^C-Phe as precursor (24). Internal validation of this single biopsy approach showed an analytical intra-day repeatability (*n* = 6 days) of FSR of 6%.

### Plasma Amino Acid Analysis

Within 30 min following blood sampling, the samples were centrifuged at 1,800 g for 20 min at +4°C (3,200 rpm). About 500 μl of the supernatant were transferred into a fresh 1.5 mL Eppendorf tube and frozen at −80°C.

Samples were thawed and then work on ice at ~4°C. The samples were centrifuged for 1 min at 8,000 rpm at 4°C. The 200 μl aliquot was then transferred to a fresh Eppendorf tube. While vortexing, 20 μl of ice cold (2–8°C) sulfosalycilic acid 40% solution were added to the plasma aliquot. This was then vortexed for 1 min at 1,800 rpm. The samples were then kept between 2 and 8°C for 15 min before centrifuging for 3 min at 13,000 g at 4°C.

Individual amino acid concentrations were measured by ion exchange chromatography with spectrophotometer detection after ninhydrine derivatization using and Amino Acid Analyser (Biochrom 30, Onken, Germany). The sum of all individual AA measured provided the total of plasma amino acids concentration. The sum of all individual essential AA's measured, provided the essential plasma AA concentration.

### Western Analysis of AKT/p70S6k Signaling Proteins

Western blot analysis was performed using the NuPage System precast gels as described by the manufacturer (Novex, Life Technologies). About 40 mg of frozen muscle tissue was homogenized in ice cold lysis buffer A (50 mmol/L Tris-HCL, pH 7.5, 1 mmol EDTA/L, 1 mmol EGTA/L, 10%glycerol, 1%triton-X, 50 mmol NaF/L, 5 mmolNa4P2O7/L, 1 mmol dithiothreitol/L, 10 mg trypsin inhibitor/mL, 2 mg aprotinin/mL,1 mmol benzamidine/L, and 1 mmol phenylmethylsulfonylfluoride/L) using a TissueLyser (Qiagen). The lysate was centrifuged (12,000 × g, for 20 min at 4°C) and the supernatant was transferred to fresh Eppendorf tubes for subsequent quantification of protein content. The protein concentration for each muscle extract was determined using the Pierce BCA Protein Assay Kit (Thermo Scientific). Equal amounts (5 μg) of protein samples were denatured by boiling for 5 min. Subsequently, samples were loaded onto 4–12% Bis-Tris midi gels before being transferred onto nitrocellulose membranes (iBlot gel Transfer Stacks, Life Technologies) and blocked for 1 h with Odyssey Blocking Buffer at room temperature. Each membrane was then incubated overnight at 4°C with a primary antibody [Anti-AKT1 phospho (1:2,000) ab66138; Anti-rpS6 (1:1,000) ab40820; Anti-AKT1 (1:1,000) ab91505 were purchased from Abcam; Phospho-p70S6K (1:500) #9205; Phospho-rpS6 (1:1,000) #2211; Phospho mTOR (1:500) #2971; mTOR (1:1,000) #2972; α-Tubulin (1:1,000) #3873 were purchased from Cell Signaling Technology], diluted in 0.1% Tween Odyssey Blocking Buffer PBS (LI-QOR Biosciences). The following morning each membrane was washed with PBS containing 0.1% Tween 20 (PBST). The membranes were then incubated for 1 h at room temperature in a black box with IRDye800CW-conjugated goat anti-rabbit or anti-mouse secondary antibodies (LI-COR Biosciences) diluted in Odyssey Blocking Buffer. The blots were then washed four times with PBST and rinsed with PBS. Proteins were visualized by scanning the membrane on an Odyssey Infrared Imaging System (LI-COR Biosciences) with 800-nm channels.

### Insulin and Glucose Analysis

#### Insulin

Plasma insulin concentrations was measured using the commercially available IBL Insulin Enzyme Immunoassay Kit (IBL International) according to manufacturer's directions.

#### Glucose

Plasma glucose concentrations were assessed using the Siemens dimension EXL 200 clinical chemistry system (Siemens Healthcare Diagnostics AG, Zurich, Switzerland).

### Statistical Analysis

No sample size calculation have been performed due to absence of data on mean difference and variations of endpoints in this study.

Endpoints with a single measure (changes in whole body protein, FSR, signaling pathways and other variables) -were analyzed using a paired *t*-test or a Wilcoxon rank-sum test, depending on the presence of outliers (outliers were defined as value exceeding the distance from the mean by more than two standard deviations). For parameters with longitudinal measurements, a two-way ANOVA with repeated measurements was employed to test the treatment effect over time and its interaction with time. Pairwise comparisons at different time points were only evaluated in the case when the overall treatment effect was statistically significant. These rules were agreed upon in the statistical analysis plan prior to unblinding of the study. In addition, for data with 5 time points, mean profile of insulin, glucose and amino acids was estimated using spline and summarized by an AUC. Estimates of tmax (time where concentration is maximum), Cmax (value of the maximum concentration), AUC (area under the curve), and AUCc (area under the curve, corrected for baseline value) were based on 1,000 replicates of the mean smoothing curves in each treatment group. No multiplicity adjustments were performed. Data are expressed as means ± SDs. Significance was set at α = 0.05. The analyses were performed in R software (version 3.0.1, R Foundation for Statistical Computing).

## Results

### Baseline Characteristics

All participants in both supplementation groups in the present study had similar baseline values for age and body mass index (Table 1).

### Plasma Amino Acid Concentrations

**Figure 3:**
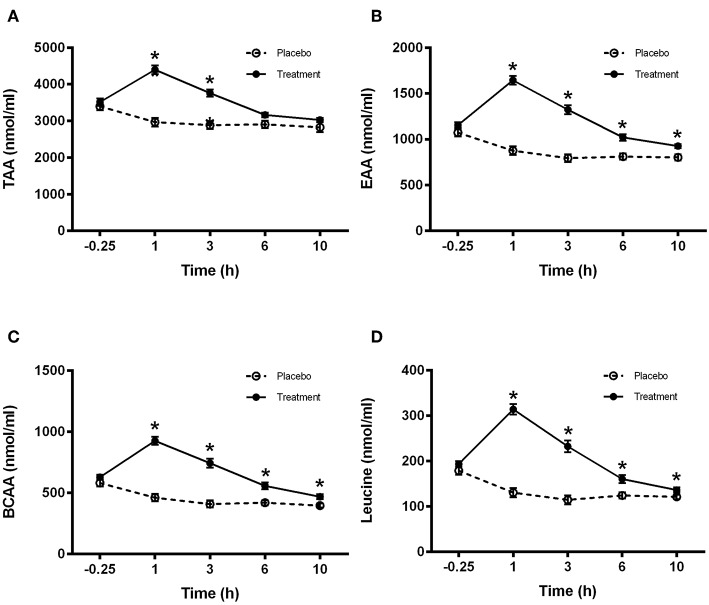
Plasma amino acid concentrations for **(A)** total (TAA), **(B)** essential (EAA), **(C)** branched chain (BCAA), and **(D)** leucine for control (light bars) and treatment (dark bars) groups observed over 10 h post supplement ingestion. Supplement was ingested immediately after the first blood sample was obtained. Values are means ± SD. ^*^Significantly different at corresponding time point between groups (*P* < 0.05).

No treatment effect was observed for either total amino acids, essential amino acids, branched chain amino acids or leucine plasma concentrations between groups at baseline. However, total amino acid plasma concentrations were significantly greater in the treatment group compared to the placebo group at 1 and 3 h (*P* < 0.001; Figure 3A). Essential amino acid plasma concentrations were significantly higher in the treatment group compared to placebo at 1 h (*P* < 0.001), 3 h (*P* < 0.001), 6 h (*P* < 0.001), and 10 h (*P* = 0.01) (Figure 3B). Furthermore, branched chain amino acid plasma concentrations were significantly higher in the treatment group compared to placebo at 1 h (*P* < 0.001), 3 h (*P* < 0.001), 6 h (*P* < 0.01), and 10 h (*P* = 0.01) (Figure 3C). Finally, leucine plasma concentrations were significantly greater in the treatment group compared to placebo at 1 h (*P* < 0.001), 3 h (*P* < 0.001), 6 h (*P* = 0.01), and 10 h (*P* = 0.05) (Figure 3D).

### Plasma Insulin and Glucose Concentrations

**Figure 4:**
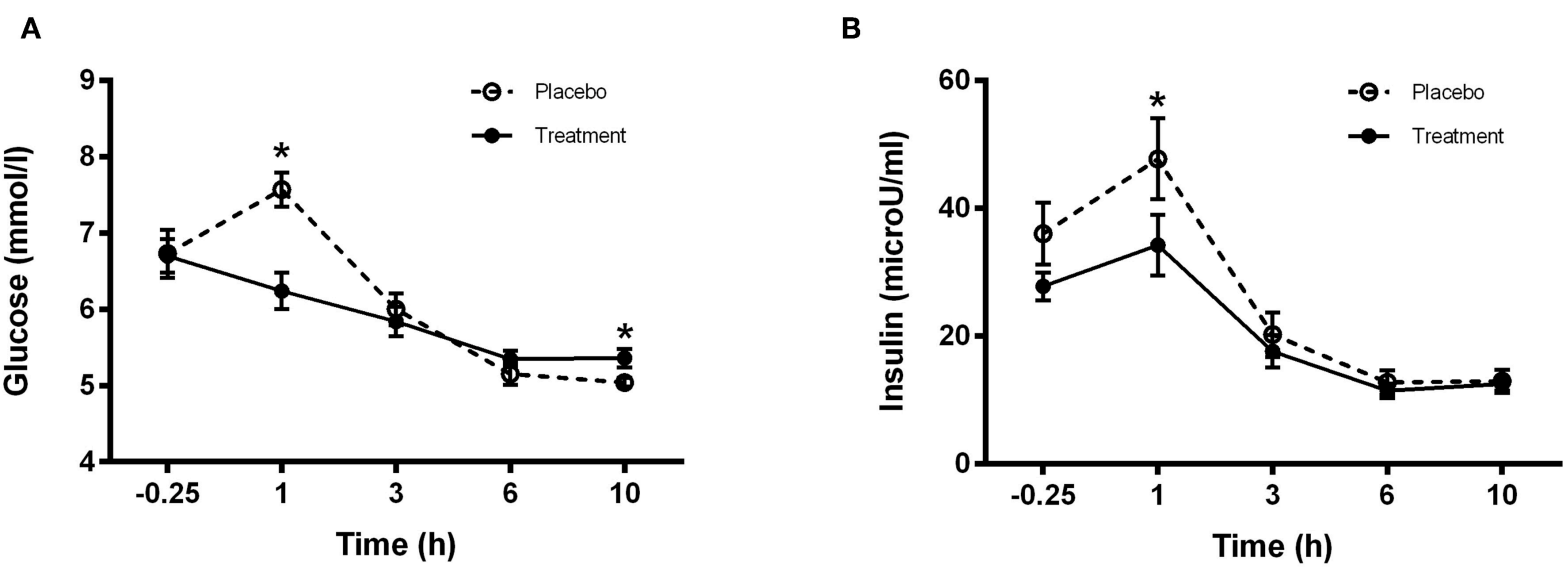
Plasma **(A)** Glucose and **(B)** Insulin concentrations for control (light bars) and treatment (dark bars) groups observed over 10 h post supplement ingestion. Supplement was ingested immediately after the first blood sample was obtained. Values are means ± SD. ^*^Significantly different at corresponding time point between groups (P < 0.05).

Plasma insulin and glucose concentrations are presented in Figures 4A,B, respectively. A treatment effect was observed at time 1 h for insulin showing a Cmax significantly higher (*P* < 0.05) in the placebo group compared to treatment (Figure 4B). A significant difference was observed in the glucose concentrations between treatments at 1 h (placebo group higher) and 10 h (treatment group higher) (*P* < 0.001 and *P* < 0.05, respectively; Figure 4B).

### Rates of Muscle Protein Synthesis

**Figure 5:**
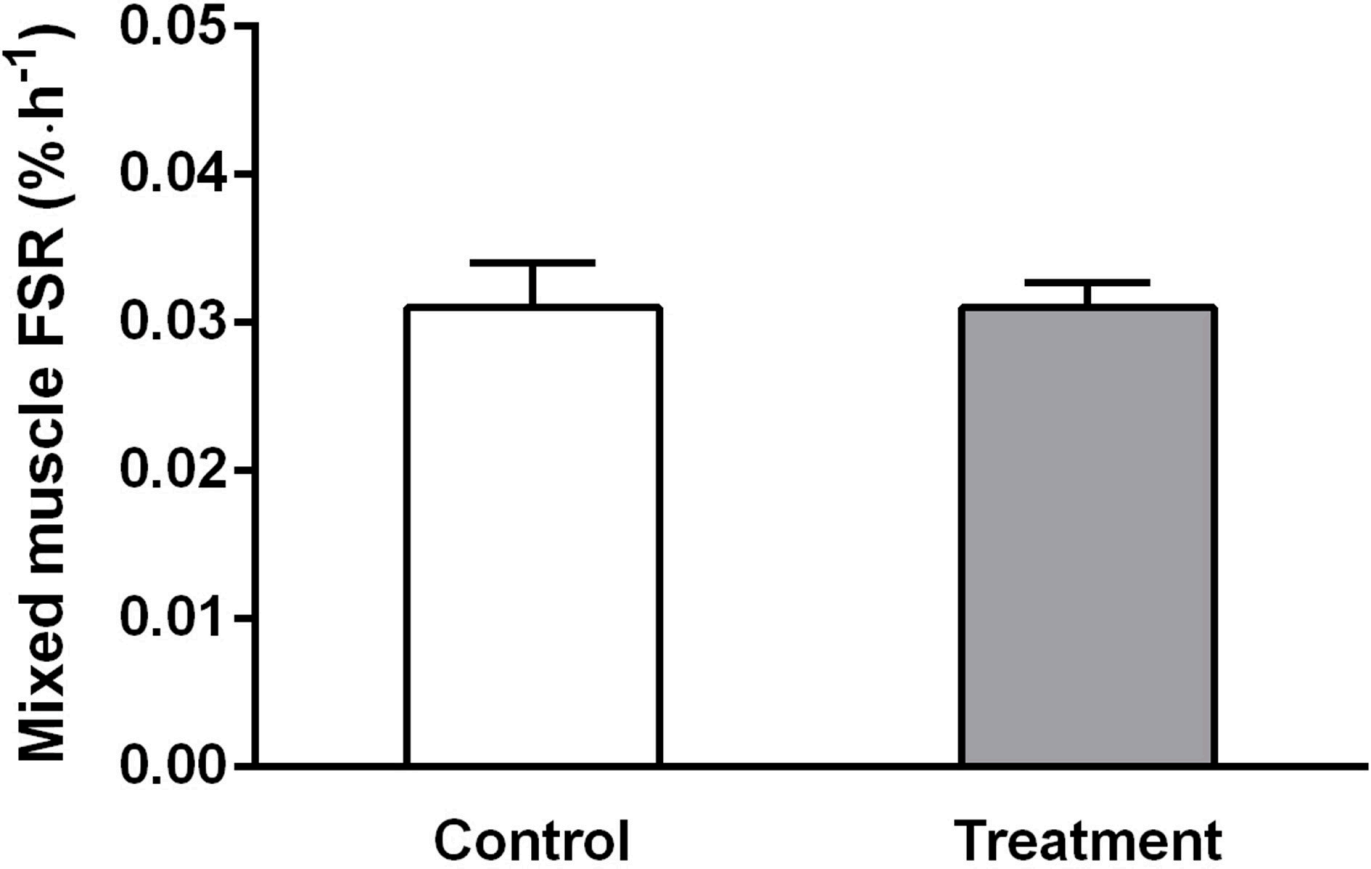
Mixed muscle rates of protein synthesis measured between 03:00 and 08:00 h in control (light bars) and treatment (dark bars) groups. Values are means ± SD. No significant difference was observed between treatments.

Plasma free phenylalanine enrichment was significantly different between pre-administration of the tracer and 1 h after (7 h post-drink, data not reported). When plotting the average TTR of free ^13^C-Ph in plasma over time (7, 10, and 11 h), the slope was not significantly different from 0 (*P* = 0.17). TTR ranged from 0.0702 ± 0.006 at 7 h to 0.0673 ± 0.0082 (*n* = 17). These data suggest that plasma free ^13^C-Ph reached a plateau and subjects were in isotopic steady state condition throughout the duration of the tracer infusion. The rates of muscle protein synthesis were measured between 03:00 and 08:00. No significant changes were observed in the rate of muscle protein synthesis, over time or between treatments (Figure 5).

### Whole Body Protein Balance

**Figure 6:**
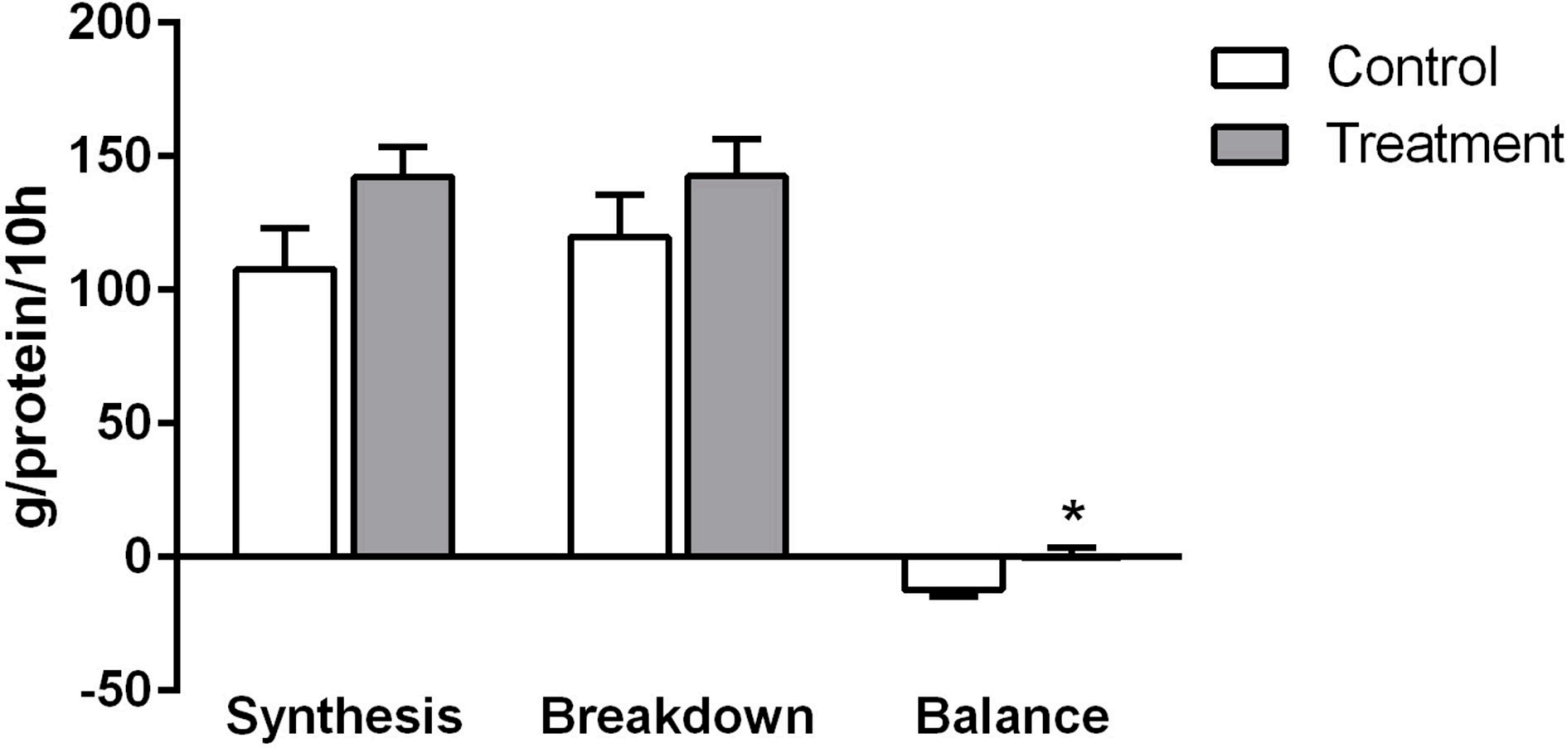
Rates of whole body protein synthesis, breakdown and balance in control (light bars) and treatment groups (dark bars) observed across the 10 h overnight sleep duration. Values are expressed as means ± SD. To test the treatment effect over time, a two-way ANOVA with repeated measurements was employed. Pairwise comparisons at different time points were only evaluated in case when overall treatment effect was statistically significant, ^*^Significantly different from control (P < 0.05).

The ingestion of the placebo drink resulted in negative whole body protein balance across the entire 10 h overnight sleep period (−12.22 g prot/10 h). In contrast, the ingestion of the protein containing drink resulted in a near zero protein balance across the entire 10-h overnight sleep period (−0.13 g prot/10 h) and this difference was significantly different compared to the placebo group (*P* < 0.01) (Figure 6). One individual in the placebo group had to be excluded because it was not possible to obtain a muscle sample. No significant differences were observed in either whole body protein synthesis or breakdown (Figure 6).

### Western Analysis of Muscle Signaling Proteins

**Figure 7:**
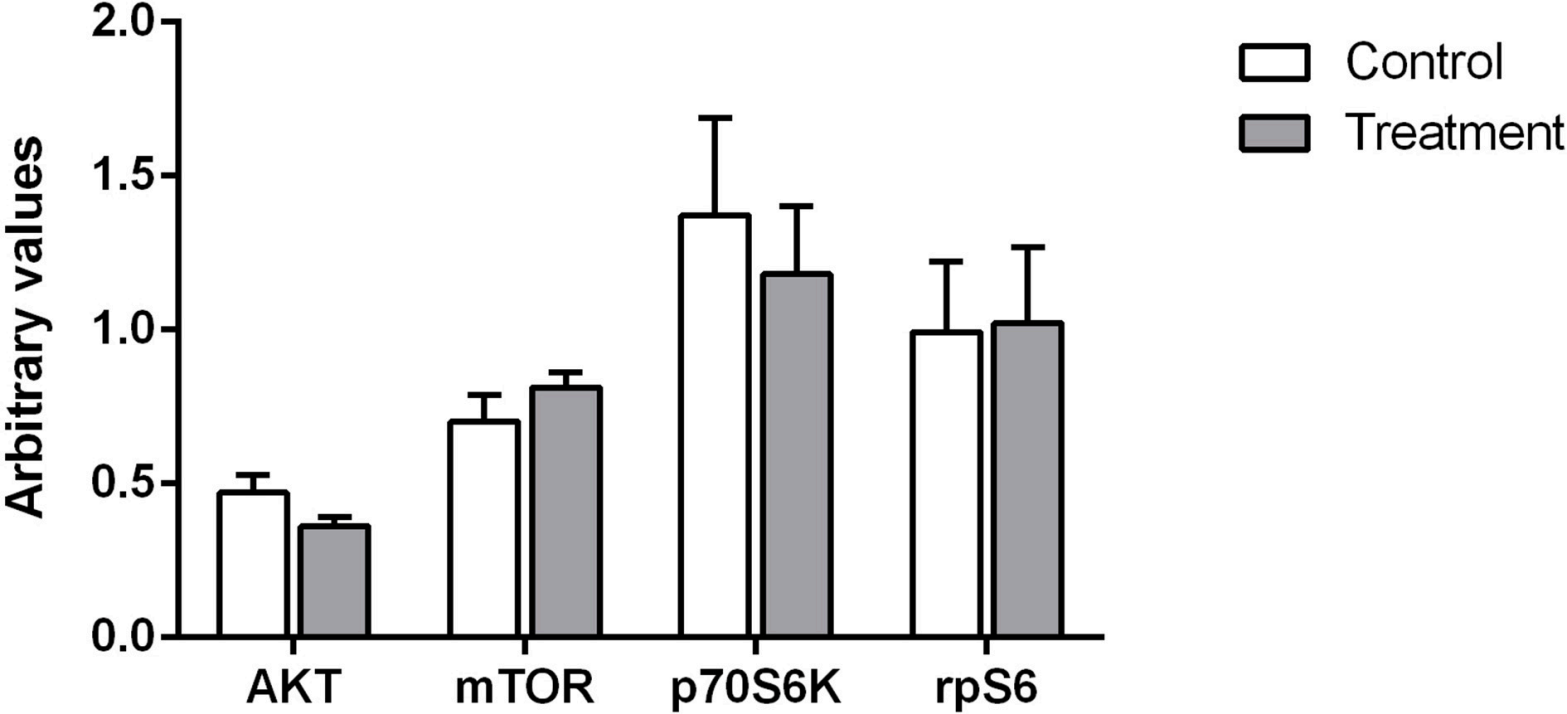
Relative phosphorylation status of proteins involved in skeletal muscle protein synthesis for AKT, mTOR, p70S6K, and rpS6. Values are expressed as means ± SD and are expressed as the ratio between phosphorylated forms to total protein and corrected for a house keeper. No significant differences were observed for any of the proteins between treatments.

Western analysis was carried out on the muscle biopsy obtained at 08:00. No significant changes were observed between treatments for any of the signaling proteins examined (Figure 7).

## Discussion

To date, only a few studies have investigated the impact of pre-bedtime dietary protein ingestion on either whole body or muscle specific protein turnover and most of this work has been carried out as part of a resistance exercise training program in young and elderly healthy physically active individuals (17, 18, 25–28).

In light of the accelerated loss of lean tissue mass observed in the elderly (including muscle), nutritional strategies aimed at preventing such losses are of key importance in such populations. A recent paper showed that the ingestion of a 40 g protein bolus before sleep increased muscle protein synthesis during overnight sleep (19). In our present study, we report that providing a complete protein containing drink (containing 25 g of dietary protein together with 25 g carbohydrate and 7.5 g oil) to healthy middle-aged individuals before sleep, prevented the negative whole body protein balance and that muscle protein synthesis specifically, is not augmented in the later part of the night despite elevated aminoacidemia.

Interestingly Res et al., previously reported that a total of 60 g of casein resulted in greater whole body protein balance and increased muscle protein synthesis in young exercising participants (18) while Groen et al. showed that nasogastric feeding of 40 g casein commencing at 02:00 in elderly resulted in normal digestion and absorption kinetics, stimulating muscle protein synthesis and improved overnight whole body protein balance (29). In these studies, muscle protein synthesis was measured over the whole night and thus it is unknown if the effect observed was related to a stimulation of protein synthesis occurring all along the night in response to the sustained amino acid availability or only during the first hours after protein ingestion (as usually described when a meal effect is studied).

In our study we still observed amino acid digestion kinetics similar to those previously reported in individuals throughout the night. Specifically, we observed substantial elevations in plasma amino acid concentrations (TAA, BCAA, EAA, Leucine) throughout the night when compared to the isocaloric, protein void control drink which is even more extended than previously reported by Kouw et al. (19) who reported a return to baseline values about 6 h following the ingestion of 20 g whey protein. In our study, we still observed higher aminoacidemia values in the treatment group after 10 h which may in part be due to either a slower digestion rate related to the presence of carbohydrate and fat in our treatment drink, the additional 5 g of protein provided in our treatment drink or a combination of both. Moreover, given that muscle protein synthesis is only stimulated for several hours following an increase in plasma amino acid concentrations (5, 30, 31) it is not clear if the observed benefits in whole body protein balance in our study as well as the increased rates of muscle protein synthesis and whole body net protein balance observed previously by others were driven at specific time points (0–4 h following protein ingestion) or across the whole duration of the night (extended amino acid availability).

Accordingly, Kouw et al. concluded that the greater plasma amino acid availability they observed following 40 g of protein ingestion compared to 20 g during the longer overnight period may have been required in order to allow a measurable increase in muscle protein synthesis rates. To address this question we assessed muscle protein synthesis at the later part of the night between the hours of 03:00–08:00. Despite the extended increases in plasma amino acid concentrations observed in our study (Figure 3) we found that rates of muscle protein synthesis at the later part of the night did not differ between groups. In addition we also quantified the activation of the canonical signaling cascade implicated in skeletal muscle protein synthesis and found no significant differences between treatment groups for either AKT, mTOR, rpS6, or p70S6K (Figure 7). In light of these observations, it seems therefore that the observed increase in whole body protein balance in our study was most likely driven by the immediate postprandial response to the beverage ingestion (first 4 h post-ingestion).

What is not clear however, is why the extended and elevated amino acid plasma concentrations did not stimulate muscle protein synthesis. One possibility is that the threshold plasma levels of circulating amino acids required to stimulate muscle protein synthesis was not reached. Indeed, when we compared the activation of the mTOR signaling pathway in the morning at waking we did not observe any changes between treatments in relative phosphorylation of either mTOR, rpS6, or p70S6K which are known to be activated by amino acid availability (9). In the absence of changes in the rates of muscle protein synthesis at the later part of the sleep period as well as the lack of observed changes in the anabolic signaling pathway, we propose that any observed change in whole body protein balance was likely due to the postprandial effect of protein ingestion (<4 h following ingestion) or that the improved whole body protein balance was primarily driven by other organs other than muscle. The former is partly supported by a recent study in elderly individuals in which the authors investigated the effect of a bolus vs. a pulse feeding pattern of amino acids on muscle protein synthesis. They reported extended elevated essential aminoacidemia in the absence of increased muscle protein synthesis (32) which the authors attributed to the “muscle full” state (a state describing the point at which stimulation of muscle protein synthesis becomes refractory to the ongoing availability of EAA).

The insulin response to the treatment and placebo beverages displayed significantly different values at the 1 h time point whereby the placebo ingestion resulted in a greater insulin peak compared to the treatment group (Figure 4B). This is not surprising given the increased maltodextrin content of the placebo beverage (in order to provide isocaloric properties between treatment and placebo groups). Furthermore, ingestion of the placebo beverage also resulted in a higher glucose peak compared to the protein group 1 h post ingestion. However, despite the lower levels of maltodextrin in the treatment group, plasma glucose levels in the treatment group failed to return to basal levels in the morning (08:00) and therefore, fasting plasma glucose remained higher relative to the control group but no significant effect was observed for insulin. The exact physiological significance of this response however is not clear. Several studies have reported mixed observations on measures of glucose handling in the morning following either pre-bedtime or overnight nutrient provisions (27, 29, 33–35). In a study investigating the effect of providing a pre-bed time protein or carbohydrate snack to overweight sedentary women, the authors reported that plasma insulin concentrations were negatively affected resulting in an increased homeostatic model assessment of insulin resistance score indicating an insulin resistant phenotype (33). In contrast, introducing physical activity to the daily routine prevented the negative responses in insulin (34). It seems therefore that beyond the caloric content, the habitual physical activity status of an individual is an important determinant regulating the metabolic milieu following a pre-bedtime nutrient containing snack. In addition, the overall body composition of an individual may also further impact the results, as lean tissue percentage may impact insulin sensitivity and/or glucose uptake, something we were not able to control for in the present work. However, one limitation in the current study which prohibits us to conclude on the impact of pre-bed protein ingestion on the subsequent morning glycemic control is the absence of a fasting baseline glucose measure. This would have enabled us to better compare fasting glycemia pre and post-intervention.

Moreover, it is important that the circadian rhythm and an integrative physiology approach should be considered when planning pre-bedtime nutritional strategies aimed at promoting specific health outcomes (36–39). For instance controlling for sleep time as well as meal compositions 2–3d prior to an intervention seems ever more important in order to account for circadian rhythm disturbances which may in turn impact the results. Whether these short term benefits are translated into long term functional outcomes remains to be seen. Nonetheless, in a recent study it was shown that long-term pre-bedtime protein supplementation in healthy young men resulted in greater gains in both, strength and muscle mass following a 12 week progressive resistance training program (27). However, what is not clear is whether or not this effect was due to the timing of the protein ingestion *per se* (before bed) or simply due to the extra 25 g of protein ingested above that of the control group in a 24 h period.

In conclusion we show that the ingestion of a complete drink containing 25 g of milk protein before sleep promotes whole body protein balance in the morning compared to the ingestion of an isocaloric, protein void pre-bed supplement in middle-aged healthy men. Moreover, this effect was not related to a stimulation in the rates of skeletal muscle protein synthesis in the second part of the night, suggesting the observed anabolic effect was likely a typical postprandial response observed in the first half of the sleep period. Such nutritional strategies may be implemented in order to attenuate age related losses of lean tissue mass but other metabolic outcomes such as glucose handling need to be incorporated and assessed to prevent any disturbances to the metabolic milieu. Better understanding of the role of chronobiology and how it is impacted by nutrition is of critical significance in promoting long-term benefits of nutrient timing interventions for the promotion and maintenance of health and well-being.

## Data Availability Statement

The datasets generated for this study are available on request to the corresponding author.

## Ethics Statement

The studies involving human participants were reviewed and approved by Commission Cantonale d'Ethique (VD) pour la Recherche sur l'Etre Humain, Switzerland, under the reference 423/13. The patients/participants provided their written informed consent to participate in this study.

## Author Contributions

LK conceptualized the research. LK, DB, MB, J-PG, and MS designed the research. LD-C and KR-K were responsible for product development. DD, SP, A-FK, and JV performed the analysis. MS was responsible for the statistical analysis and reporting. LK and DB were responsible for data interpretation. LK wrote the paper and had primary responsibility for final content. All authors read and approved the final manuscript.

### Conflict of Interest

LK, MB, LD-C, J-PG, A-FK, DD, SP, JV, MS, KR-K, and DB were employees of Nestlé Research at the time the study was carried out.
